# The Acute Effects of Caffeinated Black Coffee on Cognition and Mood in Healthy Young and Older Adults

**DOI:** 10.3390/nu10101386

**Published:** 2018-09-30

**Authors:** Crystal F. Haskell-Ramsay, Philippa A. Jackson, Joanne S. Forster, Fiona L. Dodd, Samantha L. Bowerbank, David O. Kennedy

**Affiliations:** 1 Brain, Performance and Nutrition Research Centre, Northumbria University, Newcastle Upon-Tyne NE1 8ST, UK; philippa.jackson@northumbria.ac.uk (P.A.J.); jo.forster@northumbria.ac.uk (J.S.F.); f.dodd@northumbria.ac.uk (F.L.D.); david.kennedy@northumbria.ac.uk (D.O.K.); 2 Faculty of Health and Life Sciences, Northumbria University, Newcastle Upon-Tyne NE1 8ST, UK; samantha.bowerbank@northumbria.ac.uk

**Keywords:** coffee, caffeine, chlorogenic acids, phenolic, cognition, cognitive, mood, age, sex

## Abstract

Cognitive and mood benefits of coffee are often attributed to caffeine. However, emerging evidence indicates behavioural effects of non-caffeine components within coffee, suggesting the potential for direct or synergistic effects of these compounds when consumed with caffeine in regular brewed coffee. The current randomised, placebo-controlled, double-blind, counterbalanced-crossover study compared the effects of regular coffee, decaffeinated coffee, and placebo on measures of cognition and mood. Age and sex effects were explored by comparing responses of older (61–80 years, *N* = 30) and young (20–34 years, *N* = 29) males and females. Computerised measures of episodic memory, working memory, attention, and subjective state were completed at baseline and 30 min post-drink. Regular coffee produced the expected effects of decreased reaction time and increased alertness when compared to placebo. When compared to decaffeinated coffee, increased digit vigilance accuracy and decreased tiredness and headache ratings were observed. Decaffeinated coffee also increased alertness when compared to placebo. Higher jittery ratings following regular coffee in young females and older males represented the only interaction of sex and age with treatment. These findings suggest behavioural activity of coffee beyond its caffeine content, raising issues with the use of decaffeinated coffee as a placebo and highlighting the need for further research into its psychoactive effects.

## 1. Introduction

Coffee consumption is associated with a number of health benefits in elderly men and women including reduced risk of cardiovascular disease (CVD) [1], lower incidence of type 2 diabetes mellitus [2], and decreased death from inflammatory diseases [3], CVD [4,5], and all-cause mortality [6,7]. A number of physiological factors associated with these conditions are relevant to cognitive function in healthy ageing, as well as pathological ageing conditions such as dementia or Alzheimer’s disease (AD). Indeed, a number of epidemiological studies have demonstrated an association between higher coffee consumption and better performance on cognitive tests in older adults [8,9], as well as an inverse relationship between coffee consumption and risk of dementia/AD [10,11,12,13,14].

Cognitive benefits from coffee consumption are typically attributed to caffeine, which exerts its effects through non-selective antagonism of adenosine A_1_ and A_2A_ receptors [15]. In support of this, a number of studies have demonstrated the ability of caffeine to improve measures of attention and increase ratings of alertness [16,17,18]. However, coffee contains more than 1000 different compounds including phenolics, diterpenes, and melanoidins [19], all of which have the potential to affect behaviour either directly or indirectly through interaction with caffeine. This is demonstrated by studies showing direct psychoactive effects and modulation of caffeine’s effects by the amino acid l-theanine, present in tea [20,21,22]. Similarly, lengthened startled blink onset latency has been shown following decaffeinated coffee as compared to caffeinated coffee, caffeinated juice, and non-caffeinated juice [23]. Chlorogenic acids (CGA) are a group of phenolic compounds representing the principal non-caffeine components in coffee [24] and have been explored in relation to mood and cognition in healthy, elderly participants [25]. In comparison to regular CGA decaffeinated coffee (224 mg CGA, 5 mg caffeine), high CGA decaffeinated coffee (521 mg CGA, 11 mg caffeine) increased alertness and decreased negative emotional processing, whereas caffeinated coffee (244 mg CGA, 167 mg caffeine) increased accuracy on a sustained attention task and improved mood. These results indicate that the addition of CGA to regular decaffeinated coffee can modulate its effects on behaviour. The effects of CGA were explored further in a study comparing 540 mg isolated CGA, 6 g decaffeinated green blend coffee (532 mg CGA), and placebo [26]. Whilst positive effects on mood were observed following decaffeinated green blend coffee, these effects were not evident following CGA in isolation, which also led to detrimental effects to cognition at 120 min post-drink. This provides further evidence for behavioural effects of decaffeinated coffee and highlights the need to consider the synergistic contribution of non-caffeine compounds in coffee.

Coffee is one of the most widely consumed beverages in the world, yet intervention trials examining the specific impact of consuming regular, brewed coffee on cognition and mood are lacking. Given the potential for non-caffeine components within coffee to exert psychoactive effects or to interact synergistically with caffeine, it is important that the effects of regular coffee and decaffeinated coffee are compared to placebo. In addition, despite physiological differences between men and women, including in their nutrient needs and in cognitive performance [27,28], sex differences are rarely considered in nutritional intervention trials. This is particularly important here, as studies of the relationship between coffee and cognitive decline have indicated that whilst reduced risk is related to coffee consumption in men [29], the effect is more pronounced in women [30,31]. This suggests that greater effects of coffee consumption may be observed in older adults as a consequence of cognitive decline, and that these beneficial effects may be enhanced in females. Furthermore, given the impact of the menstrual cycle on resting metabolic rate [32] and systemic clearance of caffeine [33], it is also possible that sex differences in response will be moderated by age. In order to explore this further, the current study compared the behavioural effects of regular coffee, decaffeinated coffee and placebo in elderly participants (61–80 years) to those in a younger (20–34 years) adult group and examined differential responses in men and women. As debate continues as to whether caffeine’s effects are modulated by habitual consumption [34,35] only those who regularly consumed coffee and tea were included.

## 2. Materials and Methods

### 2.1. Design

A randomised, placebo-controlled, double-blind, counterbalanced-crossover design was employed. The study was approved by Northumbria University’s Faculty of Health and Life Sciences Ethics Committee (reference: SUB057_Forster_090216; approved: 26 February 2016) and was conducted in accordance with the Declaration of Helsinki.

### 2.2. Participants

Seventy-two participants were drawn through an opportunity sample within Newcastle upon Tyne and the surrounding areas. Thirty-six of these represented an older group aged 61 to 80 years (18 male), and 36 represented a younger comparator group aged 20 to 34 years (18 male). Sample size was determined from a power calculation based upon previous data showing improvements to cognition and mood in habitual caffeine consumers following 150 mg caffeine [16]. An effect size of *d* = 0.6 indicated that a total of 72 participants would allow detection of significant effects with a power of 0.8. All participants were healthy non-smokers for whom English was their first language. Participants were not currently taking medication with the exception of contraception in young female participants and those used in the treatment of arthritis, high blood pressure, high cholesterol, and reflux-related conditions in the older participant group. Due to the potential impact of habitual caffeine intake on response, only those who regularly consumed more than two cups of coffee or three cups of tea (equating to ≥150 mg caffeine/day) were included. Participants were paid £60 for taking part.

**Figure 1:**
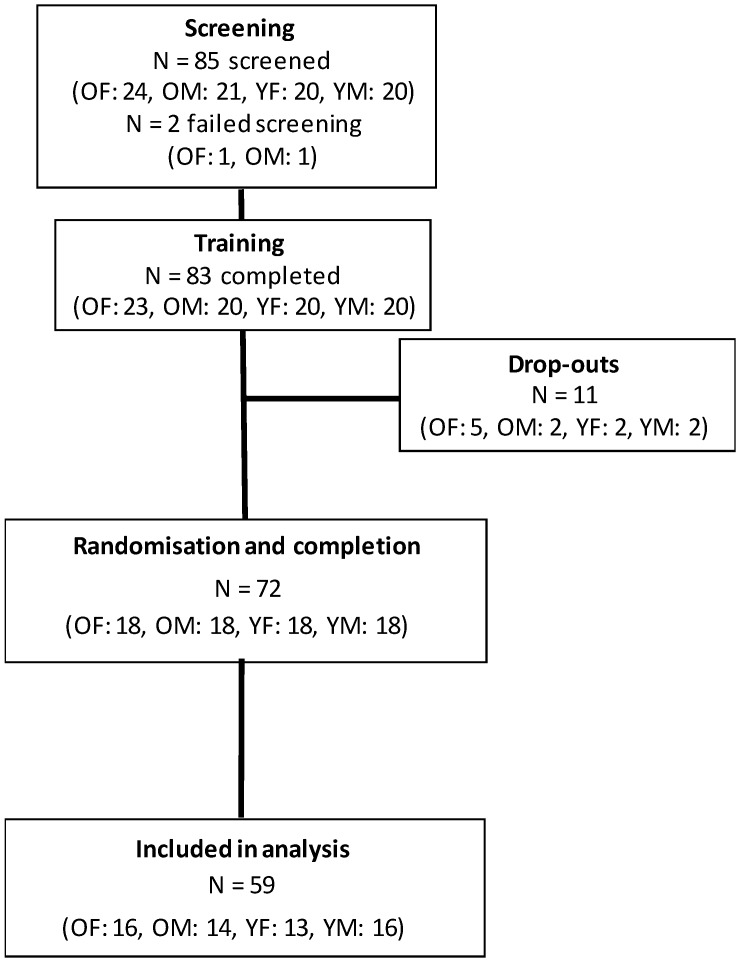
Final participant disposition. N = Number of participants; OF = Older Female; OM = Older Male; YF = Younger Female; YM = Younger Male.

Thirteen participants were excluded from the per protocol analysis (12 based on high (>1 µg/mL) pre-dose caffeine salivary levels, and one due to under-consumption of the drink provided). The population for analysis (see Figure 1) consisted of 30 older adults (14 male) and 29 young (16 males). Participant characteristics for each age group by sex can be found in Table 1.

### 2.3. Treatment

At each study visit, one of the following drinks was administered by an independent third party with no further involvement in the study.

- 220 mL water mixed with 2.5 g coffee flavouring (placebo)
- 220 mL regular coffee (without milk and sugar) containing 100 mg caffeine
- 220 mL decaffeinated coffee (without milk and sugar) containing ~5 mg caffeine

The order in which participants received each drink was determined by computer-generated random allocation (Latin square) for each sex (male, female) by group (older and younger age comparators). Regular and decaffeinated coffee were brewed using two separate drip filter coffee makers following a standardised brewing procedure, including the use of filter papers to minimise cafestol and kahweol levels. Placebo consisted of 2.5 g flavouring (maltodextrin 2.26 g, dark roast 0.1 g, mild roast 0.1 g, and coffee natural 0.04 g—Firmenich SA, Meyrin, Satigny, Switzerland) added to boiling water. Drinks were matched for temperature (58 °C) and served in an opaque thermal beaker with a black opaque straw with 5 min allowed for drinking.

### 2.4. Salivary Caffeine Levels

Saliva samples were obtained using salivettes (Sarstedt, Leicester, UK). Samples were taken immediately prior to baseline assessments in order to confirm compliance to abstinence and following post-drink assessments to confirm effective caffeine absorption. The saliva samples were immediately frozen at −20 °C until thawing. Once thawed, salivette tubes were centrifuged at 15,000× *g* for 10 min. Stock solutions of caffeine, paraxanthine, and benzotriazole (internal standard) were prepared in type I ultra-pure water at a concentration of 100 µg/mL. Calibration standards for caffeine and paraxanthine were prepared between 0.05 and 5.00 µg/mL. Quality control samples were also prepared at a concentration of 2.5 µg/mL. Internal standard (50 µL at 5.0 µg/mL) was added to 50 µL of each standard and sample in duplicate. To extract the compounds 2 mL of ethyl acetate was added and solutions were vortex mixed for 3 min following by centrifugation at 4000× *g* for 10 min. The organic layer was transferred to a clean tube and dried under a stream of nitrogen at 45 °C. The residue was reconstituted in 100 µL of mobile phase and 50 µL injected onto the column.

Saliva samples were analysed with high-performance liquid chromatography. The HPLC system was an Agilent 1260 Infinity™ (Cheadle, Greater Manchester, UK) consisting of an Infinity™ quaternary pump, an Infinity™ Autosampler with integrated column oven and an Infinity™ multi-wavelength detector set at 280 nm. Instrument control and data processing was performed using Agilent OpenLab™ CDS (Agilent Technologies Ltd., Cheadle, UK). Chromatographic separation was achieved on a Kinetex C_18_ column (4.6 × 250 mm i.d., particle size 5 µm; Phenomenex Ltd., Macclesfield, UK). The mobile phase consisted of acetonitrile, acetic acid and type I ultra-pure water (5:1:95, *v*%:*v*%:*v*%) delivered at a flow rate of 1.00 mL/min.

### 2.5. Cognitive and Mood Measures

With the exception of driving ability, all cognitive and mood measures were delivered using the Computerised Mental Performance Assessment System (COMPASS, Northumbria University, Newcastle upon Tyne, UK), a purpose-designed software application for the flexible delivery of randomly generated parallel versions of standard and novel cognitive assessment tasks. This assessment system has previously been shown to be sensitive to nutritional interventions [36,37] including caffeine [20]. The tasks and mood scales were chosen based on their known sensitivity to caffeine or susceptibility to ageing. Tasks were presented in the same order on each occasion and, with the exception of the paper and pencil tasks (immediate and delayed word recall and verbal fluency), responses were made using a response pad. The entire selection of tasks took approximately 25 min to complete. See Table 2 for order and scoring of tasks completed at baseline. Due to the potential for interference from repeat completions, computerised location learning and driving simulation were only completed post-dose on study visits with the final session from their training day used as the statistical baseline in the analyses.

### 2.6. Caffeine Research Visual Analogue Scales 

Prior to cognitive assessment, subjective state was assessed with the Caffeine Research Visual Analogue Scales [38], which have previously been used in caffeine research [16,21,39]. The following descriptors are presented on-screen: ‘relaxed’, ‘alert’, ‘jittery’, ‘tired’, ‘tense’, ‘headache’, ‘overall mood’, and ‘mentally fatigued’. Participants are asked to rate how much these descriptors match their current state by placing an ‘x’ on a line with the end points labelled ‘not at all’ (left hand end) and ‘extremely’ (right hand end); with the exception of ‘headache’, which is labelled ‘no headache’ and ‘extreme headache’; and ‘overall mood’, which is labelled ‘very bad’ and ‘very good’. Ratings are scored as % along the line from left to right.

### 2.7. Computerised Location Learning—Learning Phase

Location learning was assessed with a computerised task modified from Kessels et al. [40]. Participants are shown a grid containing pictures of objects. Following a timed delay they are shown an empty grid and asked to relocate the objects to the correct location shown to them previously. In the current study, this was repeated five times during the learning phase, with objects presented for 15 s, a gap of 10 s before the empty grid was shown, and a pause of 5 s between each trial. For each of the five learning trials, a displacement score is calculated as the sum of the errors made for each object (calculated by counting the number of cells the object had to be moved both horizontally and vertically in order to be in the correct location) from each trial. A learning index is also calculated as the average relative difference in performance between trials [((A − B)/A + (B − C)/B + (C − D)/C + (D − E)/D)/4].

### 2.8. Computerised Location Learning—Delayed Trial

During the delayed trial, which took place 30 min after completion of the learning phase, participants are again asked to place the objects in the correct location on the empty grid with no further prompting. The delayed trial is scored for displacement and delayed recall, which is calculated as the difference between displacement score on the final learning trial and the delayed trial.

### 2.9. Driving Ability

A PC based driving simulation (Driving Simulator 2013, Excalibur Publishing Limited, Banbury Oxfordshire, UK) was used to assess driving ability. Driving was controlled via a steering wheel and pedals with gears set to fully automatic. The task lasted for 3 min and is scored on the basis of adhering to road rules and driving ability. Specifically, the task is scored for errors, which are given when deviating too much from the track; deviating too much from the instructed directions; not indicating; speeding; colliding. If the drive ended (either because of collision or because of exceeding 10 errors) the task was restarted but no more than two restarts (three drives in total) were allowed.

### 2.10. Procedure

**Figure 2:**
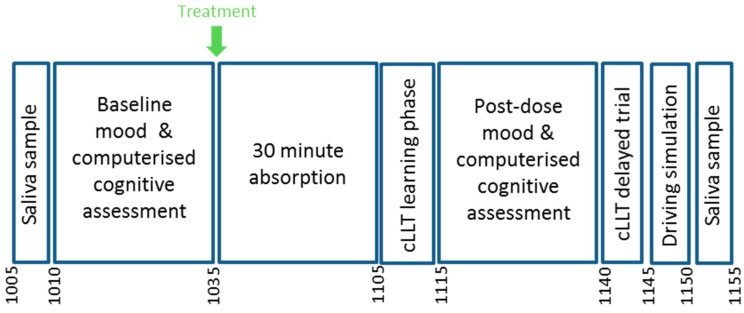
Study visit timeline.

Potential participants attended the Brain Performance and Nutrition Research Centre at Northumbria University for an initial screening session where they gave informed consent prior to participation. Their eligibility was assessed in accordance with the criteria outlined in the ‘Participants’ section and training on the computerised tasks was provided. This consisted of five completions of cognitive tasks and took place on a single day. Participants attended three study visits, separated by at least seven days to allow for washout and to prevent confound due to caffeine abstention instructions. These instructions required abstention from caffeine from noon the day before study visits but this did not exceed 24 h in order to minimise any potential withdrawal effects. Consumption of alcohol and over-the-counter medication was also restricted for 24 h (48 h in the case of systemic antihistamines). On the morning of study visits, participants ate their usual breakfast at least 1 h prior to arrival at the laboratory with the time and composition of breakfast standardised across visits. Participants attended the laboratory at 9:45 a.m. and were screened to ensure eligibility for testing that day, this included checking they were in good health and had adhered to instructions regarding breakfast consumption, and caffeine, alcohol, and medication restrictions. A food diary was used to aid with breakfast standardisation and a saliva sample was obtained to confirm adherence to caffeine abstention instructions. All testing took place in a suite of dedicated temperature-controlled university laboratories with participants visually isolated from each other and wearing noise-reduction headphones to decrease the impact of any auditory distractions. Baseline assessments of cognition and mood were completed and participants were then given their drink for that day. After 30 min of rest in the laboratory, the learning phase of a computerised Location Learning Test (cLLT) was completed before parallel versions of the tasks completed at baseline. This was followed by the delayed trial of the cLLT, a driving simulation task and a final saliva sample for assessment of caffeine levels. At the end of the final visit only, participants were asked to guess which drink they believed they had consumed that day. See Figure 2 for a schematic depicting the study visit running order.

### 2.11. Statistics

The post-dose outcome measures were modelled using the MIXED procedure in SPSS (version 24.0, IBM Corp., Armonk, NY, USA) which included the respective baseline values and the terms treatment, age, sex, treatment × age, treatment × sex and treatment × age × sex as fixed factors. In the case of computerised location learning and driving simulation, baseline values were taken from the final training session. Significant effects were followed up with Bonferroni corrected pairwise comparisons.

## 3. Results

### 3.1. Treatment-Related Effects

#### 3.1.1. Salivary Caffeine

**Figure 3:**
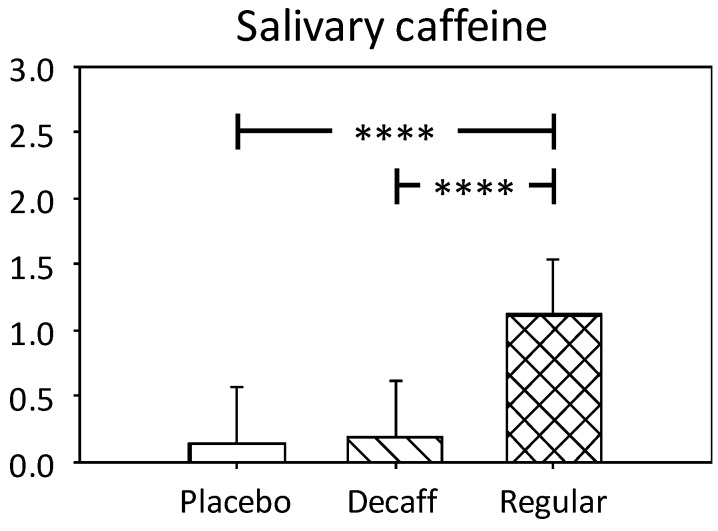
Adjusted means + standard error for salivary caffeine measured in µg/mL. Significant treatment effect **** *p* < 0.001.

Baseline salivary caffeine values were 0.17 µg/mL, confirming adherence to caffeine abstention instructions. A significant main effect of treatment was observed on post-dose salivary caffeine (F(2, 101.1) = 155.6, *p* < 0.0001). Pairwise comparisons revealed significantly greater levels following caffeinated coffee compared to placebo (*p* < 0.0001) and decaffeinated coffee (*p* < 0.0001). See Figure 3.

#### 3.1.2. Digit Vigilance

**Figure 4:**
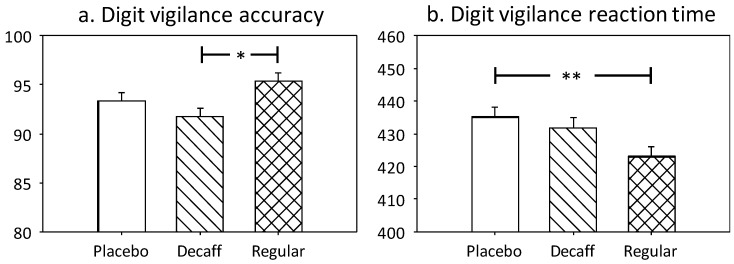

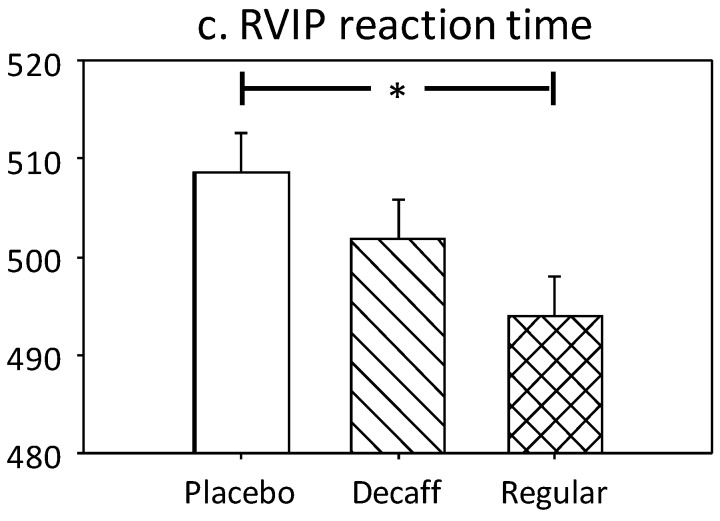
Adjusted means + standard error for those cognitive measures showing significant effects of treatment. (**a**) Digit vigilance accuracy; (**b**) Digit vigilance reaction time; (**c**) Rapid Visual Information Processing (RVIP) reaction time. Accuracy is measured as % and reaction time in milliseconds. * *p* < 0.05; ** *p* < 0.01.

A significant main effect of treatment was observed for digit vigilance accuracy (F(2, 101.1) = 4.44, *p* = 0.014). Pairwise comparisons revealed significantly greater accuracy following regular coffee compared to decaffeinated coffee (*p* = 0.01). See Figure 4a.

Digit vigilance reaction time was also significantly affected by treatment (F(2, 71.3) = 5.07, *p* = 0.009). Pairwise comparisons revealed significantly faster responses following regular coffee compared to placebo (*p* = 0.009). See Figure 4b.

#### 3.1.3. Rapid Visual Information Processing

Rapid visual information processing reaction time showed a significant effect of treatment (F(2, 102.9) = 3.77, *p* = 0.026). This was due to significantly faster responses following regular coffee compared to placebo (*p* = 0.02). See Figure 4c. A significant treatment x sex interaction was also observed for false alarms (F(2, 93.3) = 4.55, *p* = 0.013) but pairwise comparisons revealed no significant effects.

#### 3.1.4. Computerised Location Learning Delayed Trial

Computerised location learning recall showed a significant treatment x sex interaction (F(2, 104) = 3.46, *p* = 0.035). However, pairwise comparisons revealed no significant effects.

#### 3.1.5. Alert

**Figure 5:**
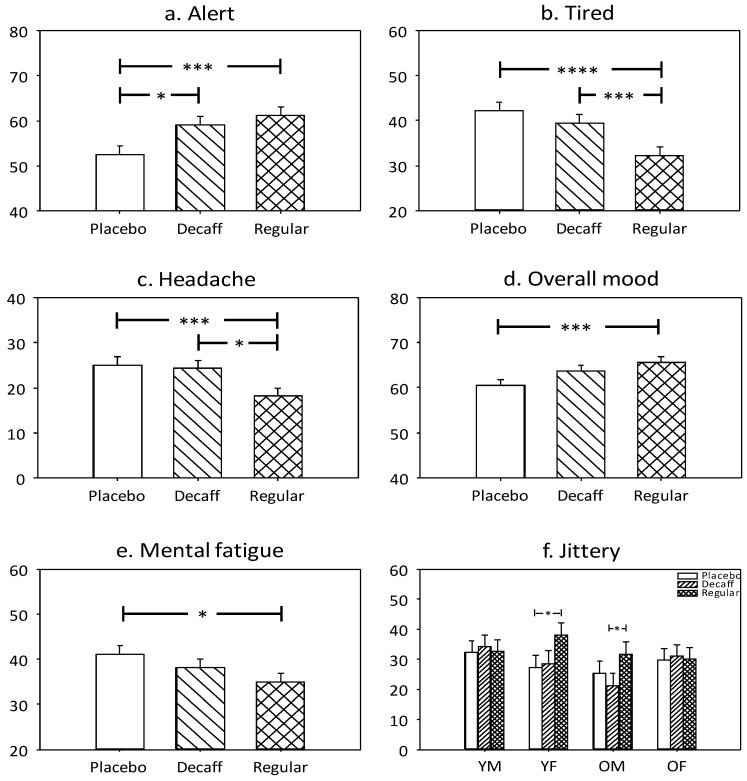
Adjusted means + standard error for those mood measures showing significant treatment-related effects. (**a**) Alert; (**b**) Tired; (**c**) Headache; (**d**) Overall mood; (**e**) Mental fatigue; (**f**) Jittery. Ratings are measured as % along a visual analogue scale with higher values indicating greater response. YM = young male; YF = young female; OM = older male; OF = older female; * *p* < 0.05; *** *p* < 0.005; **** *p* < 0.001.

Ratings of ‘alertness’ were significantly affected by treatment (F(2, 106) = 9.86, *p* < 0.0001). This was due to significantly higher ratings following regular coffee (*p* < 0.0005) and decaffeinated coffee (*p* = 0.0048) compared to placebo. See Figure 5a.

#### 3.1.6. Tired

A significant main effect of treatment on ‘tired’ ratings was also observed (F(2, 101.4) = 12.31, *p* = 0.0001). Pairwise comparisons revealed this was due to significantly lower ratings following regular coffee compared to decaffeinated coffee (*p* = 0.003) and placebo (*p* < 0.0001). See Figure 5b.

#### 3.1.7. Headache

A significant main effect of treatment on headache ratings (F(2, 92.9) = 6.31, *p* = 0.003) was due to significantly lower ratings following regular coffee compared to decaffeinated coffee (*p* = 0.0049) and placebo (*p* = 0.015). See Figure 5c.

#### 3.1.8. Overall Mood

‘Overall mood’ was significantly affected by treatment (F(2, 105.8) = 5.56, *p* < 0.005). This was due to significantly higher ratings following regular coffee compared to placebo (*p* = 0.004). See Figure 5d.

#### 3.1.9. Mental Fatigue

A significant main effect of treatment on ‘mental fatigue’ ratings was also observed (F(2, 97.5) = 4.43, *p* = 0.014). Pairwise comparisons revealed this was due to significantly lower ratings following regular coffee compared to placebo (*p* = 0.01). See Figure 5e.

#### 3.1.10. Jittery

A significant treatment × age × sex interaction was observed on jittery ratings (F(3, 76.2) = 3.01, *p* = 0.035). Pairwise comparisons revealed significantly higher ratings following regular coffee compared to placebo in young females (*p* = 0.046) and compared to decaffeinated coffee in older males (0.045). See Figure 5f.

Unadjusted means, standard deviations, and *F* and *p* values for all factors (treatment, age, sex) and their interactions can be found in Tables S1–S3.

### 3.2. Treatment Guess

Seventy-one percent of participants correctly guessed which drink they had received at the final visit. Eighty-one percent correctly guessed they had received regular coffee, 72% correctly identified decaffeinated coffee, and 58% were able to correctly identify placebo as their final drink.

## 4. Discussion

Consumption of 220 mL of regular coffee containing 100 mg caffeine led to faster responses during digit vigilance and rapid visual information processing tasks when compared to placebo, and to increased digit vigilance accuracy when compared to decaffeinated coffee. In terms of mood effects, ratings of alertness and overall mood were higher and mental fatigue ratings lower following regular coffee compared to placebo. Tiredness and headache ratings were lower following regular coffee compared to placebo and decaffeinated coffee. Rating of jitteriness was the only outcome to show an interaction with sex and age indicating higher ratings following regular coffee when compared to placebo in young females and when compared to decaffeinated coffee in older males. Decaffeinated coffee also engendered an increase in subjective alertness, compared to placebo, whereas accuracy of digit vigilance and tired and headache ratings were impaired in comparison to regular coffee. A beneficial effect of decaffeinated coffee was also observed following a treatment × age × sex interaction, which indicated that ratings of jitteriness were significantly lower following decaffeinated compared to regular coffee in older men. The pattern of response to decaffeinated coffee generally fell between responses to regular coffee and placebo. Specifically, numeric working memory accuracy and reaction time, reaction time for attention tasks and mood ratings, all followed the order of placebo > decaffeinated > regular coffee (see Supplementary Tables), with the exception of relaxed and tense ratings which showed a preferential effect of decaffeinated coffee.

The findings with regards regular coffee are largely in line with the reported effects of caffeine, which only has a consistent beneficial effect on attention task performance and subjective alertness/arousal [16,17,18]. Whilst this could be taken as support for the notion that caffeine is the sole contributor to the effects, the finding of psychoactive effects of decaffeinated coffee, in terms of increased alertness when compared to placebo and a pattern of lower effects than regular coffee in comparison to placebo, supports the suggestion of a modulatory role for the non-caffeine compounds within coffee. The effects of decaffeinated coffee presented here are broadly in line with previous results showing impairment to accuracy of a sustained attention task in comparison to regular coffee [25] and increases in alertness when compared to placebo and the phenolic acid CGA in isolation [26]. Previous studies have highlighted CGA as a potentially important component of coffee. However, whilst there is some evidence for beneficial modulation of coffee’s effects by increasing CGA content [25], the effects of CGA in isolation were largely negative [26]. This potentially highlights an issue in applying a reductionist approach to nutritional interventions where complex interactions between many different components may be required to see optimum results. Although composition is varied depending on roasting and brewing techniques, caffeine generally only accounts for ~1% and CGA ~10% of the weight of coffee beans, this leaves almost 90% of the constituents unaccounted for. It is also important to note that the CGA profile may be altered as part of the decaffeination process and therefore any analysis of effects must take account of the impact of decaffeination on other constituents [41].

The observed benefits for regular coffee are expected due to the known effects of caffeine in antagonising adenosine A_1_ and A_2A_ receptors thereby, increasing oxygen metabolism [42] and upregulating various neurotransmitters including noradrenaline, dopamine, serotonin, acetylcholine, glutamate, and GABA [15]. Caffeine and its metabolites also have a number of mechanistic properties that make them liable to have a modulatory or interactive effect when caffeine is co-consumed with other bioactive compounds. These include the inhibition of enzymes involved in the breakdown of neurotransmitters (e.g., acetylcholinesterase and monoamine oxidase) and cellular signalling molecules (e.g., phosphodiesterase and PARP (poly(ADP-ribose)polymerase)) [43,44] and a role as a competitive substrate for a number of cytochrome P450 (CYP) enzymes (CYP2A1, CYP2E1, and CYP1A1) that metabolise endogenous and exogenous chemicals in the human body [45,46,47]. Of particular relevance here, low-doses of caffeine have therefore been shown to increase the bioavailability of phenolic compounds [48,49,50] and have a synergistic effect in terms of the cardiovascular benefits of polyphenols.

Coffee also has the potential to impact glucose metabolism as evidenced by an increase in insulin sensitivity observed following decaffeinated coffee when compared to placebo [51]. Interestingly, this effect was not apparent following regular coffee, which may be due to counteractive effects of caffeine and non-caffeine components within regular coffee. Support for this comes from data showing decreased insulin sensitivity following caffeine [52]. Moreover, area under the curve (AUC) profiles for serum insulin indicate that caffeine increases AUC when compared to decaffeinated coffee and placebo, whilst regular coffee produced a trend towards the same when compared to decaffeinated coffee, with similar profiles evinced for glucose AUC [53]. CGA derivatives have been shown to increase insulin sensitivity in rats [54], and further support for the role of phenolic compounds in this effect comes from data showing modulation of glucose and insulin response following phenolic-rich berries [55,56,57] as well as a reduction in the postprandial blood glucose response following grape seed extract [58]. Similarly, caffeine is known to have a vasoconstrictive effect, including reduced cerebral blood flow (CBF) [39], whereas phenolic-rich foods have demonstrated the opposite effect. Of particular relevance is the ability of phenolic-rich cocoa to increase CBF when compared to a phenolic-poor control matched for methylxanthine content [59,60]. These findings indicate the ability of coffee components to counteract the negative effects of caffeine and a potential synergy whereby phenolic compounds increase CBF, and therefore oxygen supply, whilst caffeine increases brain activity and subsequent oxygen metabolism. It is also possible that caffeine increases absorption of phenolics as has been shown following consumption of cocoa [48] but is as yet untested following coffee.

A further consideration is that due to a focus on psychoactive effects of caffeine, the cognitive and mood effects of coffee have typically been measured at 30 to 120 min post-dose coinciding with a peak in caffeine levels at around 40-min post-ingestion [61]. However, analysis of the fate of CGA following coffee consumption shows that whilst a number of phenolic acids and their derivatives peak between 30 and 60 min, others do not appear until between 4- and 6-h post-ingestion [62]. It is therefore necessary to extend the testing period in order to fully examine the impact of these metabolites. This is also true in relation to caffeine, which has a half-life of around 5 h [63], and has demonstrated behavioural effects up to 8 h post-dose [64]. It is therefore probable that any effects of coffee observed at 6-h would represent an interaction between phenolic acids and caffeine and the measurement of biomarkers would aid in elucidating the role of each.

Learning and episodic memory tasks showed the expected effect in terms of significantly lower performance in the older cohort when compared to young (see Supplementary Tables). However, no interactions between age and treatment were observed on any cognitive measure. Whilst learning and memory are not typically susceptible to caffeine, it has been proposed that these tasks may show sensitivity in low arousal situations as is expected in the elderly as energetic resources diminish [65]. However, in the current study there was no evidence of higher arousal in the young sample when compared to the older participants on subjective measures of ‘arousal’ or psychomotor tasks. This may suggest that the older cohort studied here were relatively high functioning, as is supported by their status being higher than national averages both in relation to fruit and vegetable consumption and education level [66]. It has also been suggested that cognitive benefits of caffeine consumption may be more pronounced in those aged over 80 years [31,67]. Therefore, the findings reported here do not preclude interaction effects in an older sample with poorer nutritional status and/or lower education level.

Similarly, although sex differences were observed in the current study, these did not interact with treatment as may have been expected from data showing greater benefits of coffee consumption in women than men [30]. However, the potential mechanisms underlying sex differences following habitual consumption, including sex steroid levels [68,69], haemodynamic mechanisms [70], and uric acid responses [71,72], are unlikely to exert effects over a 30-min time period. Furthermore, given the impact of the menstrual cycle and hormonal contraception on metabolism, it is possible that any differential sex effects in the younger cohort were obscured by the lack of control for menstrual cycle phase and the inclusion of four hormonal contraceptive users in this study. This also potentially explains large variations in salivary caffeine following regular coffee in young females that were not observed in the young male group. Similar large variations in response were shown for older men and women indicating individual differences in response to caffeine. This variability is largely due to differences in CYP1A2 activity, which is influenced by a number of factors including sex and genetic polymorphisms [73].

Polymorphisms of the ADORA2A gene may only explain the age, sex, treatment interaction, which indicated that older men and younger women experienced greater feelings of jitteriness following regular coffee. It has previously been reported that those who are T/T homozygous at nucleotide positions 1976C > T and 2592Tins experience increases in anxiety after caffeine administration that are not observed in the other genotypic groups [74,75]. Moreover, sex differences in response have been noted for 1976TT homozygotes, whereby females are more susceptible to anxiogenic effects of caffeine than males [76,77]. Interestingly, whilst there is evidence for reduced caffeine intake in 1976TT homozygotes [78], others have shown that intake of coffee, but not other sources of caffeine, is increased. It was also shown that increased habitual consumption moderated anxiogenic effects of caffeine such that they were only observed in non/light consumers of caffeine, irrespective of genotype [79]. This indicates that even in those with a genetic predisposition, tolerance to anxiogenic effects can occur with habitual consumption. As the older men in the current study consumed less coffee than their female counterparts, this may in part explain the specificity of ‘jittery’ effects observed here. However, there are a multitude of factors that impact on interindividual differences [80], which require further exploration before definitive conclusions can be reached.

In the current study, although the addition of a true placebo builds on research previously limited to comparing effects following caffeinated and decaffeinated coffee, one important limitation is the omission of a caffeine-only arm. The inclusion of a caffeine-only condition would have allowed a direct comparison of any synergistic effects between caffeine and the other bioactive compounds in coffee, including the phenolic compounds. It may also have facilitated in blinding of drinks. Although the placebo drink in the current study was somewhat effective in that 42% incorrectly identified it, only 1 of 19 participants mistook it for regular coffee. It is important to note that this measure was only included at the end of the final visit when all three drinks had been consumed and, therefore, does not rule out the blinding of participants at earlier visits. However, as the stimulant effects of caffeine are easily detected, the inclusion of a caffeine-containing ‘placebo’ would provide an active control for regular coffee and reduce the ability of participants to correctly identify regular coffee.

The findings presented here suggest behavioural activity of coffee beyond its caffeine content. In fact, only one cognitive measure and two subjective measures showed significant differences between regular and decaffeinated coffee in favour of regular coffee. This highlights two key issues with studies which compare regular and decaffeinated coffee. Firstly, these studies attribute any differential effects to caffeine without considering the potential for interaction with other components. Secondly, any synergistic effects of caffeine and other coffee components within regular coffee are likely to be underestimated due to the potential for behavioural effects of decaffeinated coffee used as the control. If the effects of regular coffee are to be fully understood, it is important that future research compares these to the equivalent dose of caffeine, decaffeinated coffee, and placebo. Furthermore, research in this area must include plasma levels of potentially important compounds, including phenolic compounds. This would allow assessment of the impact of caffeine on the pharmacodynamic profile of other components in coffee. An extended testing period is also recommended in order to capture effects of colonic metabolites of phenolics appearing at ~8 h. Further research is also required in which cognition is measured alongside potential underlying mechanisms including, but not limited to, glucoregulation and modulation of cerebral haemodynamics. Finally, in order to capture the impact of interindividual differences in metabolism of caffeine and other components of coffee on behavioural outcomes, genetic factors should also be considered.

## Figures and Tables

**Table 1:** Participant demographics (SD = Standard Deviation).

	Young				Older			
	Male		Female		Male		Female	
	Mean	SD	Mean	SD	Mean	SD	Mean	SD
Age	26.3	4.4	26.2	3.6	67.7	6.3	67.1	3.4
Years in education	18	3	17	3	16	5	14	4
Body Mass Index (BMI)	25.7	3.8	23.8	3.6	25.9	3.4	26.1	3.9
Caffeine consumption (mg/day)	327	88.2	351	110.4	426	74.4	394	87.8
Coffee consumption (cups/day)	2.88	1.54	2.54	1.45	2.64	1.13	3.59	0.93
Fruit and vegetables (portions/day)	4.3	1.5	4.1	1.4	4.4	1.9	5.4	1.8

**Table 2:** Cognitive tasks completed at baseline and 30 min post-dose in order of presentation (computerised location learning and driving ability are described below).

Task	Descriptor	Scoring	Domain
Word presentation	A series of words is displayed on the screen, one word at a time. In this case, 15 words were presented with a display time of 1 s and interstimulus interval of 1 s	-	
Immediate word recall	Participants are instructed to write down the words that were presented. In this case, 60 s were given to complete the task	Number correct and number of errors	Episodic memory
Picture presentation	A series of photographic images are displayed on the screen, one at a time. In this case, 15 images were presented with a display time of 2 s and an interstimulus interval of 1 s	-	
Simple reaction time	An upwards pointing arrow is displayed on the screen at irregular intervals. Participants must respond as quickly as they can as soon as they see the arrow appear. In this case, 50 stimuli were presented	Reaction time (ms)	Attention
Digit vigilance	A fixed number appears on the right of the screen and a series of changing numbers appear on the left of the screen at the rate of 150 per minute. Participants are required to make a response when the number on the left matches the number on the right. In this case the task lasted for 3 min	Accuracy (%), reaction time for the correct responses (ms) and false alarms (number)	Attention
Numeric working memory	Five single target numbers are displayed on the screen, one at a time. Participants are required to memorise these numbers as they appear. Once the target series has been presented, numbers are displayed one at a time and participants are required to indicate if each number was presented in the previous list or not. In this case, three trials were completed	Accuracy (%) and reaction time for the correct responses (ms)	Working memory
Verbal fluency	Participants are presented with a letter on screen and asked to write down as many words as they can, beginning with that letter. In this case, the letters presented were A, T, C, F, M, and S and 60 s were given to complete the task	Number correct permitted words, with names and perseverations discounted from the total score	Language
Delayed word recall	Participants are instructed to write down the words that were presented to them at the beginning of the assessment. In this case, 60 s were given to complete the task	Number correct and number of errors	Episodic memory
Rapid visual information processing	A continuous series of single digits are presented in the centre of the screen at the rate of 100 per minute. Participants are required to make a response when three consecutive odd or three consecutive even digits are displayed. In this case, the task lasted for 5 min, with eight correct target strings presented in each minute.	Accuracy (%), reaction time for the correct responses (ms) and false alarms (number)	Attention
Delayed word recognition	All target words that were shown during Word presentation plus an equal number of decoys are displayed on the screen one at a time. Participants indicate if they remember seeing the word earlier or not.	Accuracy (%) and reaction time for the correct responses (ms)	Episodic memory
Delayed picture recognition	All target pictures shown during Picture presentation plus an equal number of decoys are displayed on the screen one at a time. Participants indicate if they remember seeing the picture earlier or not.	Accuracy (%) and reaction time for the correct responses (ms)	Episodic memory

## Supplementary Materials

The following are available online at http://www.mdpi.com/2072-6643/10/10/1386/s1, Table S1: saliva; Table S2: cognition; Table S3: mood.
