# Supplementary material for: The Acute Effects of Caffeinated Black Coffee on Cognition and Mood in Healthy Young and Older Adults
Source: Nutrients. 2018 Sep 30;10(10):1386. doi: 10.3390/nu10101386 (PMC6213082; doi:10.3390/nu10101386)
Supplement: Supplementary file 1 [file nutrients-10-01386-s001.pdf]

Supplementary Table 1 - Unadjusted values for salivary caffeine levels

Task	Treatment	Age	Sex	Baseline Mean	Baseline SD	Post-dose Mean	Post-dose SD	F	p	
Salivary caffeine - µg/mL	Placebo	Young	Male	0.13	0.19	0.09	0.10			
			Female	0.24	0.28	0.25	0.32			
		Older	Male	0.16	0.15	0.09	0.10			
			Female	0.13	0.07	0.13	0.07	0.98	<0.001	Treatment
	Decaffeinated coffee	Young	Male	0.11	0.10	0.11	0.11	19.9	>0.1	Age
			Female	0.24	0.37	0.25	0.33	2.05	0.007	Sex
		Older	Male	0.19	0.16	0.16	0.11	1.21	>0.1	Treatment * Age
			Female	0.21	0.24	0.29	0.35	0.14	>0.1	Treatment * Sex
	Caffeinated coffee	Young	Male	0.08	0.12	0.84	0.21	0.38	0.064	Treatment * Age * Sex
			Female	0.18	0.28	1.36	0.64			
		Older	Male	0.22	0.26	1.15	0.61			
			Female	0.20	0.23	1.13	0.56			

Supplementary Table 2 - Unadjusted scores for cognitive tasks

Task [Domain]	Treatment	Age	Sex	Baseline Mean	Baseline SD	Post-dose Mean	Post-dose SD	F	p	
Computerised location learning displacement score [Learning]	Placebo	Young	Male	16.6	13.0	14	11			
			Female	15.4	9.7	15	8			
		Older	Male	29.5	18.7	28	16			
			Female	35.6	16.5	35	17	0.98	>0.1	Treatment
	Decaffeinated coffee	Young	Male	16.6	13.0	13	8	19.87	<0.001	Age
			Female	15.4	9.7	12	10	2.05	>0.1	Sex
		Older	Male	29.5	18.7	27	15	1.21	>0.1	Treatment * Age
			Female	35.6	16.5	32	20	0.14	>0.1	Treatment * Sex
	Caffeinated coffee	Young	Male	16.6	13.0	11	8	0.38	>0.1	Treatment * Age * Sex
			Female	15.4	9.7	18	11			
		Older	Male	29.5	18.7	24	13			
			Female	35.6	16.5	27	14			
Computerised location learning index [Learning]	Placebo	Young	Male	0.8	0.2	0.86	0.20			
			Female	0.9	0.2	0.78	0.23			
		Older	Male	0.6	0.4	0.49	0.52			
			Female	0.5	0.4	0.42	0.41	0.20	>0.1	Treatment
	Decaffeinated coffee	Young	Male	0.8	0.2	0.86	0.21	12.61	<0.001	Age
			Female	0.9	0.2	0.83	0.22	0.02	>0.1	Sex
		Older	Male	0.6	0.4	0.50	0.39	0.67	>0.1	Treatment * Age
			Female	0.5	0.4	0.49	0.24	0.30	>0.1	Treatment * Sex
	Caffeinated coffee	Young	Male	0.8	0.2	0.77	0.58	0.05	>0.1	Treatment * Age * Sex
			Female	0.9	0.2	0.79	0.33			
		Older	Male	0.6	0.4	0.54	0.36			
			Female	0.5	0.4	0.56	0.30			
Immediate word recall correct - number [Episodic memory]	Placebo	Young	Male	7.7	2.2	7.5	1.6			
			Female	6.9	1.8	7.2	1.5			
		Older	Male	5.0	1.5	5.3	1.7			
			Female	5.3	2.0	4.7	1.6	2.91	0.059	Treatment
	Decaffeinated coffee	Young	Male	7.5	2.3	7.1	1.9	21.59	<0.001	Age
			Female	7.8	1.4	6.0	1.3	6.79	0.013	Sex
		Older	Male	5.4	1.8	5.3	1.9	2.00	>0.1	Treatment * Age
			Female	5.9	2.2	4.8	1.9	0.78	>0.1	Treatment * Sex
	Caffeinated coffee	Young	Male	7.2	2.5	7.3	1.8	0.44	>0.1	Treatment * Age * Sex
			Female	7.2	1.5	6.5	1.6			
		Older	Male	5.3	1.6	4.7	1.5			
			Female	5.3	1.7	4.2	1.8			
Immediate word recall error - number [Episodic memory]	Placebo	Young	Male	0.2	0.4	0.3	0.6			
			Female	0.3	0.6	0.3	0.6			
		Older	Male	0.9	1.1	0.6	0.7			
			Female	1.0	1.0	0.8	0.8	2.52	0.085	Treatment
	Decaffeinated coffee	Young	Male	0.2	0.4	0.4	0.6	6.02	0.017	Age
			Female	0.2	0.4	0.6	0.9	2.56	>0.1	Sex
		Older	Male	1.3	1.0	0.9	1.2	0.49	>0.1	Treatment * Age
			Female	0.8	1.1	1.4	1.3	0.75	>0.1	Treatment * Sex
	Caffeinated coffee	Young	Male	0.7	0.9	0.3	0.5	0.18	>0.1	Treatment * Age * Sex
			Female	0.3	0.6	0.6	0.7			
		Older	Male	0.8	0.7	0.9	1.1			
			Female	0.8	0.9	1.2	1.2			

Simple reaction time (ms) [Attention]	Placebo	Young	Male	272.3	27.7	283.72	34.88			
			Female	263.3	30.3	273.03	34.46			
		Older	Male	302.6	49.5	314.22	41.61			
			Female	299.3	57.7	307.06	60.31	1.47	>0.1	Treatment
	Decaffeinated coffee	Young	Male	270.7	29.0	296.30	55.89	2.13	>0.1	Age
			Female	260.5	26.1	272.33	43.41	5.23	0.027	Sex
		Older	Male	292.2	29.5	303.52	38.91	1.22	>0.1	Treatment * Age
			Female	305.8	115.7	299.54	97.82	0.94	>0.1	Treatment * Sex
	Caffeinated coffee	Young	Male	270.0	23.3	277.00	29.96	0.04	>0.1	Treatment * Age * Sex
			Female	306.7	94.1	276.37	29.04			
		Older	Male	322.3	54.8	329.11	76.52			
			Female	286.4	51.4	280.91	30.06			
Digit vigilance accuracy (%) [Attention]	Placebo	Young	Male	91.7	5.5	94.86	4.64			
			Female	93.3	8.6	91.28	13.29			
		Older	Male	95.0	5.1	95.21	5.65			
			Female	95.7	5.3	92.22	10.26	4.44	0.014	Treatment
	Decaffeinated coffee	Young	Male	95.0	3.9	89.86	7.78	0.12	>0.1	Age
			Female	92.0	6.6	89.40	9.90	4.30	0.044	Sex
		Older	Male	94.0	7.2	94.19	7.99	2.45	0.092	Treatment * Age
			Female	95.6	5.4	93.78	8.53	1.83	>0.1	Treatment * Sex
	Caffeinated coffee	Young	Male	93.8	6.7	95.70	5.16	0.64	>0.1	Treatment * Age * Sex
			Female	91.5	9.0	94.19	4.01			
		Older	Male	93.2	6.0	95.39	2.94			
			Female	96.7	4.1	95.56	4.73			
Digit vigilance reaction time (ms) [Attention]	Placebo	Young	Male	425.3	37.6	432.53	34.32			
			Female	400.2	36.4	418.39	39.57			
		Older	Male	437.8	36.2	454.29	22.01			
			Female	427.2	35.0	435.10	43.59	5.07	0.009	Treatment
	Decaffeinated coffee	Young	Male	419.3	33.8	432.83	40.65	0.73	>0.1	Age
			Female	417.4	31.9	425.17	33.36	0.23	>0.1	Sex
		Older	Male	441.7	21.6	442.01	32.65	0.63	>0.1	Treatment * Age
			Female	426.3	38.5	432.88	31.45	0.16	>0.1	Treatment * Sex
	Caffeinated coffee	Young	Male	419.6	40.6	417.33	34.95	0.83	>0.1	Treatment * Age * Sex
			Female	403.0	22.8	404.98	34.42			
		Older	Male	443.5	30.3	441.35	28.21			
			Female	420.1	39.1	423.59	41.78			
Digit vigilance false alarm (number) [Attention]	Placebo	Young	Male	2.3	1.4	2	2			
			Female	1.6	1.1	1	1			
		Older	Male	3.1	2.2	2	3			
			Female	2.4	2.1	2	2	0.46	>0.1	Treatment
	Decaffeinated coffee	Young	Male	1.9	1.7	3	2	0.06	>0.1	Age
			Female	1.8	1.4	1	1	2.75	>0.1	Sex
		Older	Male	3.5	3.0	2	2	1.50	>0.1	Treatment * Age
			Female	1.9	2.0	1	1	1.79	>0.1	Treatment * Sex
	Caffeinated coffee	Young	Male	1.9	1.6	1	2	1.17	>0.1	Treatment * Age * Sex
			Female	1.4	1.1	1	1			
		Older	Male	2.8	2.7	2	2			
			Female	1.8	2.1	2	2			
Numeric working	Placebo	Young	Male	96.0	4.4	95.90	2.62			
			Female	93.8	5.1	93.68	5.73			
		Older	Male	96.6	2.9	96.83	3.92			
			Female	91.7	6.5	91.81	8.41	1.72	>0.1	Treatment

memory accuracy (%) [Working memory]	Decaffeinated coffee	Young	Male	96.0	4.5	96.88	3.33	1.35	>0.1	Age
			Female	95.4	3.7	94.36	5.20	4.53	0.041	Sex
		Older	Male	95.8	4.0	95.87	2.67	1.37	>0.1	Treatment * Age
			Female	93.6	6.8	94.31	5.50	0.04	>0.1	Treatment * Sex
	Caffeinated coffee	Young	Male	96.1	3.3	96.04	3.47	0.70	>0.1	Treatment * Age * Sex
			Female	94.4	3.6	94.27	5.79			
		Older	Male	95.6	4.3	98.18	2.25			
			Female	91.7	8.3	94.38	6.72			
Numeric working memory reaction time (ms) [Working memory]	Placebo	Young	Male	701.1	81.2	712.38	81.34			
			Female	659.0	108.2	645.88	122.07			
		Older	Male	976.3	241.3	953.32	244.81			
			Female	876.3	165.1	866.11	192.35	1.67	>0.1	Treatment
	Decaffeinated coffee	Young	Male	752.4	124.2	709.95	141.99	0.75	>0.1	Age
			Female	635.9	96.5	652.00	112.14	0.00	>0.1	Sex
		Older	Male	995.5	218.8	951.37	225.64	0.79	>0.1	Treatment * Age
			Female	848.8	188.1	851.18	208.76	0.83	>0.1	Treatment * Sex
	Caffeinated coffee	Young	Male	698.3	62.9	682.82	64.63	0.11	>0.1	Treatment * Age * Sex
			Female	658.6	128.9	641.04	130.71			
		Older	Male	965.2	185.2	900.53	211.73			
			Female	894.6	172.6	837.84	155.39			
Verbal fluency (number) [Language]	Placebo	Young	Male	11.7	2.9	13.8	2.2			
			Female	13.2	3.1	14.0	4.0			
		Older	Male	10.4	2.9	12.6	2.4			
			Female	12.2	3.1	13.4	2.5	0.01	>0.1	Treatment
	Decaffeinated coffee	Young	Male	12.1	3.2	13.8	2.2	0.88	>0.1	Age
			Female	13.2	3.5	14.6	3.0	0.00	>0.1	Sex
		Older	Male	12.1	2.5	12.8	2.9	0.59	>0.1	Treatment * Age
			Female	12.9	2.9	13.6	3.3	0.45	>0.1	Treatment * Sex
	Caffeinated coffee	Young	Male	12.1	2.5	13.9	3.2	0.10	>0.1	Treatment * Age * Sex
			Female	12.0	2.9	13.2	3.9			
		Older	Male	10.9	3.8	13.1	4.0			
			Female	11.4	2.7	13.4	2.8			
Rapid visual information processing accuracy (%) [Attention]	Placebo	Young	Male	62.7	16.2	60.2	20.2			
			Female	63.7	22.6	66.5	19.9			
		Older	Male	43.2	23.9	48.0	24.6			
			Female	65.9	19.7	67.3	22.6	2.83	0.064	Treatment
	Decaffeinated coffee	Young	Male	64.4	16.4	59.8	18.3	0.14	>0.1	Age
			Female	62.5	21.2	63.5	21.0	0.69	>0.1	Sex
		Older	Male	43.9	25.7	48.2	28.3	0.68	>0.1	Treatment * Age
			Female	68.0	14.9	66.8	24.1	0.04	>0.1	Treatment * Sex
	Caffeinated coffee	Young	Male	64.4	16.6	71.4	14.8	2.35	0.079	Treatment * Age * Sex
			Female	65.6	22.7	68.8	22.6			
		Older	Male	48.4	28.2	49.5	32.9			
			Female	62.2	23.3	69.2	17.2			
Rapid visual information processing reaction time (ms) [Attention]	Placebo	Young	Male	476.9	32.7	482.98	36.15			
			Female	471.8	46.3	481.24	37.17			
		Older	Male	532.9	54.5	522.35	46.04			
			Female	529.0	71.1	545.54	76.89	3.77	0.026	Treatment
	Decaffeinated coffee	Young	Male	497.5	29.5	487.98	44.69	6.86	0.011	Age
			Female	467.0	52.3	474.42	46.32	0.40	>0.1	Sex
		Older	Male	524.4	64.1	524.59	53.94	1.52	>0.1	Treatment * Age
			Female	535.0	71.9	529.21	59.04	1.36	>0.1	Treatment * Sex

	Caffeinated coffee	Young	Male	484.1	30.9	472.74	31.10	1.38	>0.1	Treatment * Age * Sex
			Female	474.1	57.4	452.94	45.33			
		Older	Male	516.5	41.2	513.70	39.15			
			Female	528.4	63.8	526.51	67.13			
Rapid visual information processing false alarm (number) [Attention]	Placebo	Young	Male	2.2	2.3	3	3			
			Female	3.4	4.4	1	2			
		Older	Male	4.9	5.9	4	5			
			Female	3.4	2.8	3	3	0.14	>0.1	Treatment
	Decaffeinated coffee	Young	Male	2.6	1.7	3	2	2.50	>0.1	Age
			Female	1.7	3.3	2	3	0.50	>0.1	Sex
		Older	Male	3.6	5.1	3	4	2.23	>0.1	Treatment * Age
			Female	4.0	3.2	4	3	4.55	0.013	Treatment * Sex
	Caffeinated coffee	Young	Male	3.4	3.2	2	2	1.59	>0.1	Treatment * Age * Sex
			Female	2.3	2.8	2	2			
		Older	Male	4.4	6.6	4	5			
			Female	2.4	2.2	4	3			
Delayed word recall correct (number) [Episodic memory]	Placebo	Young	Male	5.4	2.2	4.1	2.0			
			Female	4.6	1.7	3.2	2.3			
		Older	Male	3.0	2.3	2.2	1.9			
			Female	2.5	2.0	1.5	1.0	1.14	>0.1	Treatment
	Decaffeinated coffee	Young	Male	5.8	2.0	3.5	1.7	10.96	0.002	Age
			Female	5.5	2.2	2.8	2.3	2.48	>0.1	Sex
		Older	Male	3.0	1.7	2.3	1.3	2.13	>0.1	Treatment * Age
			Female	3.1	1.6	2.0	1.4	0.22	>0.1	Treatment * Sex
	Caffeinated coffee	Young	Male	5.4	1.8	3.9	1.9	0.91	>0.1	Treatment * Age * Sex
			Female	5.7	2.3	3.0	2.1			
		Older	Male	2.9	2.1	1.1	1.1			
			Female	3.4	1.9	1.6	1.6			
Delayed word recall error (number) [Episodic memory]	Placebo	Young	Male	0.6	0.6	0.7	0.9			
			Female	0.7	1.4	1.3	1.1			
		Older	Male	1.4	1.3	1.7	1.5			
			Female	1.3	1.3	1.9	1.6	0.49	>0.1	Treatment
	Decaffeinated coffee	Young	Male	0.2	0.4	0.9	0.8	3.35	0.072	Age
			Female	0.6	0.8	1.7	2.2	6.01	0.018	Sex
		Older	Male	1.9	1.3	1.9	1.8	1.52	>0.1	Treatment * Age
			Female	1.5	1.3	2.1	1.2	0.54	>0.1	Treatment * Sex
	Caffeinated coffee	Young	Male	0.9	0.9	0.8	1.3	0.80	>0.1	Treatment * Age * Sex
			Female	0.4	0.7	0.9	1.0			
		Older	Male	1.6	1.5	1.5	1.9			
			Female	1.1	1.5	2.5	1.5			
Picture recognition accuracy (%) [Episodic memory]	Placebo	Young	Male	87.9	10.5	88.33	8.35			
			Female	88.2	11.8	89.49	9.31			
		Older	Male	94.5	8.6	94.76	8.84			
			Female	97.1	3.6	95.21	4.71	0.10	>0.1	Treatment
	Decaffeinated coffee	Young	Male	90.2	9.7	91.88	5.44	6.56	0.014	Age
			Female	91.7	8.6	90.00	9.43	0.26	>0.1	Sex
		Older	Male	93.3	8.3	93.57	8.21	0.13	>0.1	Treatment * Age
			Female	98.3	3.0	96.67	4.56	0.36	>0.1	Treatment * Sex
	Caffeinated coffee	Young	Male	89.0	8.8	91.25	5.56	1.60	>0.1	Treatment * Age * Sex
			Female	92.1	6.3	88.06	9.04			
		Older	Male	93.8	7.0	94.05	7.06			
			Female	97.5	3.8	96.67	3.65			

Picture recognition reaction time (ms) [Episodic memory]	Placebo	Young	Male	748.0	97.3	741.57	97.71			
			Female	706.4	105.7	662.37	67.66			
		Older	Male	943.5	142.7	966.58	159.84			
			Female	823.2	103.8	841.40	94.64	0.33	>0.1	Treatment
	Decaffeinated coffee	Young	Male	739.3	81.2	743.00	87.74	25.18	<0.001	Age
			Female	700.8	73.9	692.10	94.36	9.95	0.003	Sex
		Older	Male	923.6	96.8	955.00	151.51	0.26	>0.1	Treatment * Age
			Female	799.0	114.5	845.84	115.64	1.54	>0.1	Treatment * Sex
	Caffeinated coffee	Young	Male	752.3	83.3	769.46	130.38	0.26	>0.1	Treatment * Age * Sex
			Female	689.0	103.8	670.53	84.91			
		Older	Male	918.2	121.3	978.97	234.24			
			Female	858.8	120.1	830.36	73.80			
Word recognition accuracy (%) [Episodic memory]	Placebo	Young	Male	77.7	8.9	77.92	11.73			
			Female	80.0	10.7	77.18	8.59			
		Older	Male	72.4	8.1	73.57	10.08			
			Female	75.8	10.8	73.54	11.38	0.90	>0.1	Treatment
	Decaffeinated coffee	Young	Male	77.3	9.3	76.04	7.91	0.68	>0.1	Age
			Female	78.3	8.0	73.33	9.43	1.85	>0.1	Sex
		Older	Male	74.0	8.4	72.86	8.26	0.28	>0.1	Treatment * Age
			Female	75.8	8.1	72.08	10.17	0.37	>0.1	Treatment * Sex
	Caffeinated coffee	Young	Male	83.1	8.4	79.38	10.05	0.43	>0.1	Treatment * Age * Sex
			Female	77.4	7.7	71.02	11.42			
		Older	Male	74.3	12.4	73.57	11.58			
			Female	76.2	8.3	73.12	7.65			
Word recognition reaction time (ms) [Episodic memory]	Placebo	Young	Male	748.4	139.6	737.93	107.79			
			Female	725.6	125.1	701.29	126.74			
		Older	Male	1060.1	182.2	1050.35	169.44			
			Female	929.0	161.0	937.51	136.27	0.23	>0.1	Treatment
	Decaffeinated coffee	Young	Male	795.4	174.1	808.41	162.71	44.17	<0.001	Age
			Female	696.9	121.6	700.36	144.09	5.29	0.026	Sex
		Older	Male	1003.4	171.9	947.71	141.21	3.05	0.052	Treatment * Age
			Female	915.1	144.9	942.25	119.39	0.90	>0.1	Treatment * Sex
	Caffeinated coffee	Young	Male	822.9	233.7	790.12	118.26	2.24	0.091	Treatment * Age * Sex
			Female	700.3	107.6	668.58	108.11			
		Older	Male	989.6	139.2	1032.38	129.53			
			Female	997.8	218.7	972.72	118.09			
Computerised location learning recall (number) (Learning)	Placebo	Young	Male	0.6	2.0	1.4	2.7			
			Female	0.2	0.6	0.2	0.6			
		Older	Male	2.3	3.8	2.4	2.8			
			Female	4.5	6.9	2.9	5.5	0.16	>0.1	Treatment
	Decaffeinated coffee	Young	Male	0.6	2.0	0.3	0.7	2.43	>0.1	Age
			Female	0.2	0.6	1.0	1.9	0.04	>0.1	Sex
		Older	Male	2.3	3.8	2.3	4.2	0.20	>0.1	Treatment * Age
			Female	4.5	6.9	2.3	3.9	0.69	>0.1	Treatment * Sex
	Caffeinated coffee	Young	Male	0.6	2.0	0.8	1.3	0.51	>0.1	Treatment * Age * Sex
			Female	0.2	0.6	1.4	3.1			
		Older	Male	2.3	3.8	1.7	2.8			
			Female	4.5	6.9	2.9	4.4			
Computerised location	Placebo	Young	Male	-0.2	0.5	-1.3	2.8			
			Female	-0.1	0.3	0.0	0.0			
		Older	Male	-0.4	1.3	-1.3	3.5			
			Female	-3.0	5.2	0.1	1.8	0.67	>0.1	Treatment

learning decay (number) (Learning)	Decaffeinated coffee	Young	Male	-0.2	0.5	-0.1	0.5	0.00	>0.1	Age
			Female	-0.1	0.3	-0.8	1.7	0.07	>0.1	Sex
		Older	Male	-0.4	1.3	-0.5	1.7	0.07	>0.1	Treatment * Age
			Female	-3.0	5.2	-0.9	3.5	3.46	0.035	Treatment * Sex
	Caffeinated coffee	Young	Male	-0.2	0.5	-0.7	1.1	0.07	>0.1	Treatment * Age * Sex
			Female	-0.1	0.3	-1.2	3.1			
		Older	Male	-0.4	1.3	-0.6	1.4			
			Female	-3.0	5.2	-1.6	4.4			
Driving simulation errors (number) [Driving ability]	Placebo	Young	Male	0.2	0.4	0.2	0.4			
			Female	0.9	0.9	0.8	1.1			
		Older	Male	1.5	1.8	1.4	2.0			
			Female	1.3	1.2	1.3	1.4	1.16	>0.1	Treatment
	Decaffeinated coffee	Young	Male	0.2	0.4	0.3	0.6	4.11	0.048	Age
			Female	0.9	0.9	0.5	0.5	1.01	>0.1	Sex
		Older	Male	1.5	1.8	0.9	0.8	0.43	>0.1	Treatment * Age
			Female	1.3	1.2	0.9	1.0	0.66	>0.1	Treatment * Sex
	Caffeinated coffee	Young	Male	0.2	0.4	0.1	0.4	0.67	>0.1	Treatment * Age * Sex
			Female	0.9	0.9	0.5	0.9			
		Older	Male	1.5	1.8	0.8	0.8			
			Female	1.3	1.2	1.4	1.7			

Supplementary table 3 - Unadjusted mood scores

Task	Treatment	Age	Sex	Baseline Mean	Baseline SD	Post-dose Mean	Post-dose SD	F	p	
Relaxed	Placebo	Young	Male	64.1	13.7	56.9	14.1			
			Female	57.8	15.1	51.0	16.5			
		Older	Male	71.4	22.7	64.2	19.1			
			Female	58.5	19.0	43.0	18.1	0.87	>0.1	Treatment
	Decaffeinated coffee	Young	Male	64.2	10.9	62.6	11.5	1.19	>0.1	Age
			Female	59.5	10.6	50.8	13.7	4.93	0.030	Sex
		Older	Male	73.4	15.2	63.7	21.1	0.75	>0.1	Treatment * Age
			Female	57.8	15.2	49.2	17.9	1.06	>0.1	Treatment * Sex
	Caffeinated coffee	Young	Male	56.4	17.2	58.1	11.3	2.04	>0.1	Treatment * Age * Sex
			Female	59.8	13.4	53.5	13.3			
		Older	Male	71.9	17.3	57.4	24.1			
			Female	55.4	14.8	47.6	17.0			
Alert	Placebo	Young	Male	53.6	16.4	51.2	13.3			
			Female	50.5	20.6	50.6	16.6			
		Older	Male	74.3	14.9	63.4	20.8			
			Female	59.3	15.5	46.0	14.3	9.86	<0.001	Treatment
	Decaffeinated coffee	Young	Male	56.1	12.3	62.4	10.1	1.08	>0.1	Age
			Female	51.5	14.7	55.1	19.1	3.01	0.088	Sex
		Older	Male	72.0	14.5	67.3	19.4	0.63	>0.1	Treatment * Age
			Female	57.8	12.0	52.4	12.9	1.86	>0.1	Treatment * Sex
	Caffeinated coffee	Young	Male	48.9	13.3	60.4	9.4	1.14	>0.1	Treatment * Age * Sex
			Female	55.5	14.9	63.2	8.2			
		Older	Male	71.4	14.3	63.4	19.9			
			Female	56.7	14.9	57.0	11.6			
Jittery	Placebo	Young	Male	25.8	13.8	32.8	19.1			
			Female	28.2	20.1	29.0	16.1			
		Older	Male	15.1	11.2	20.5	16.6			
			Female	28.8	21.0	31.7	19.6	2.98	0.055	Treatment
	Decaffeinated coffee	Young	Male	29.3	17.8	36.4	16.8	1.57	>0.1	Age
			Female	27.2	17.9	29.8	19.4	0.17	>0.1	Sex
		Older	Male	19.3	11.4	18.4	11.0	0.28	>0.1	Treatment * Age
			Female	23.6	18.5	30.6	21.3	0.23	>0.1	Treatment * Sex
	Caffeinated coffee	Young	Male	31.3	20.2	35.8	17.5	3.01	0.035	Treatment * Age * Sex
			Female	23.5	14.0	37.5	19.0			
		Older	Male	16.6	17.2	27.5	18.9			
			Female	25.9	13.6	30.6	22.0			
Tired	Placebo	Young	Male	50.3	14.5	51.7	14.8			
			Female	47.0	24.5	46.8	23.8			
		Older	Male	16.9	12.6	23.2	14.0			
			Female	38.8	22.7	44.8	17.9	12.31	<0.001	Treatment
	Decaffeinated coffee	Young	Male	47.3	18.9	43.0	13.1	0.50	>0.1	Age
			Female	46.3	20.9	44.9	28.9	0.97	>0.1	Sex
		Older	Male	23.9	17.2	27.1	17.1	2.58	0.081	Treatment * Age
			Female	39.0	20.1	41.4	20.0	1.14	>0.1	Treatment * Sex
	Caffeinated coffee	Young	Male	48.7	17.8	37.1	14.5	1.39	>0.1	Treatment * Age * Sex
			Female	47.8	22.9	32.1	17.6			
		Older	Male	27.3	20.7	26.7	17.1			
			Female	40.8	20.3	35.9	14.8			
	Placebo	Young	Male	32.4	16.0	33.9	19.0			

Tense			Female	33.2	16.9	32.7	17.8			
		Older	Male	20.3	15.9	26.5	13.8			
			Female	37.3	20.6	45.5	20.2	1.38	>0.1	Treatment
	Decaffeinated coffee	Young	Male	34.3	16.5	30.9	15.6	0.71	>0.1	Age
			Female	26.3	11.8	31.5	17.5	2.03	>0.1	Sex
		Older	Male	25.9	16.8	28.3	16.2	0.41	>0.1	Treatment * Age
			Female	37.5	20.4	39.4	20.9	0.04	>0.1	Treatment * Sex
	Caffeinated coffee	Young	Male	33.1	18.6	34.9	18.2	0.95	>0.1	Treatment * Age * Sex
			Female	32.8	16.0	38.3	18.4			
		Older	Male	25.6	14.9	32.0	21.8			
			Female	34.6	16.5	40.3	19.8			
Headache	Placebo	Young	Male	25.4	18.5	28.4	20.1			
			Female	25.8	21.8	33.8	20.0			
		Older	Male	12.9	17.7	15.1	18.6			
			Female	11.8	19.8	22.4	21.8	6.31	0.003	Treatment
	Decaffeinated coffee	Young	Male	22.4	17.2	27.1	18.1	3.35	0.073	Age
			Female	28.4	20.0	35.4	21.1	2.78	>0.1	Sex
		Older	Male	8.9	14.7	14.9	17.4	0.15	>0.1	Treatment * Age
			Female	12.0	13.2	16.9	16.6	0.49	>0.1	Treatment * Sex
	Caffeinated coffee	Young	Male	35.1	24.7	27.6	21.1	0.28	>0.1	Treatment * Age * Sex
			Female	32.5	22.2	30.6	21.1			
		Older	Male	7.6	13.3	7.9	10.4			
			Female	9.3	9.8	11.3	10.7			
Overall mood	Placebo	Young	Male	58.7	10.1	56.6	13.0			
			Female	60.2	16.2	55.6	17.5			
		Older	Male	73.6	17.3	68.3	16.7			
			Female	71.0	16.7	62.9	14.1	5.56	0.005	Treatment
	Decaffeinated coffee	Young	Male	58.4	9.8	58.4	11.3	0.72	>0.1	Age
			Female	61.7	13.2	61.5	14.1	0.04	>0.1	Sex
		Older	Male	72.4	16.9	69.5	16.4	0.60	>0.1	Treatment * Age
			Female	70.1	12.6	66.1	12.4	1.29	>0.1	Treatment * Sex
	Caffeinated coffee	Young	Male	57.4	12.7	60.5	13.3	0.16	>0.1	Treatment * Age * Sex
			Female	57.0	11.9	61.5	16.5			
		Older	Male	75.2	14.7	69.8	17.7			
			Female	69.4	12.3	68.4	13.6			
Mental fatigue	Placebo	Young	Male	33.2	16.1	46.3	14.0			
			Female	36.8	22.0	43.4	16.3			
		Older	Male	17.9	16.4	28.9	14.2			
			Female	27.6	17.8	43.1	14.6	4.43	0.014	Treatment
	Decaffeinated coffee	Young	Male	38.4	16.2	37.4	13.6	0.66	>0.1	Age
			Female	35.2	19.6	42.0	20.2	2.95	0.093	Sex
		Older	Male	23.6	19.1	31.7	18.7	1.13	>0.1	Treatment * Age
			Female	33.6	17.9	42.4	16.8	0.31	>0.1	Treatment * Sex
	Caffeinated coffee	Young	Male	41.2	16.7	38.0	13.4	1.71	>0.1	Treatment * Age * Sex
			Female	32.2	17.7	35.0	14.0			
		Older	Male	25.2	20.0	28.7	15.2			
			Female	33.0	16.9	39.6	16.7			
